# Phenotypic changes in natural populations of *Anopheles gambiae* s.l. at the onset of the long dry season in tropical savannahs of Burkina Faso, West Africa

**DOI:** 10.1051/parasite/2026010

**Published:** 2026-02-24

**Authors:** Wadaka Mamai, Karine Mouline, David Renault, Kevin Hidalgo, Kounbobr Roch Dabiré, Frédéric Simard

**Affiliations:** 1 MIVEGEC, University of Montpellier, IRD, CNRS 911 Avenue Agropolis 34090 Montpellier France; 2 Institut de Recherche en Sciences de la Santé, Direction Régionale de l’Ouest 01 P.O. Box 545 Bobo Dioulasso Burkina Faso; 3 Institut de Recherche Agricole pour le Développement (IRAD) PO Box 2123 Yaoundé Cameroun; 4 UMR 6553, University Rennes, CNRS, ECOBIO (Ecosystèmes, Biodiversité, Évolution) 263 Avenue du Général Leclerc 35042 Rennes France

**Keywords:** Malaria vectors, Aestivation, Long-distance migration, Cuticular fat deposit, Body size, Ovarian development, Energy reserves

## Abstract

In the tropical savannahs with long dry seasons, malaria mosquito populations virtually disappear after the drying up of breeding sites to reappear in large numbers at the onset of next rainy season. While aestivation and long-distance migration are proposed as key strategies enabling these vectors to persist through the dry-season, the physiological, biochemical, and morphological traits underpinning these mechanisms remain insufficiently explored, particularly under natural field conditions. This study explored seasonal changes in *Anopheles coluzzii*, *An. gambiae*, and *An. arabiensis* at the onset of the dry season in the harsh savannahs of Burkina Faso, West Africa. Late-instar immature specimens were collected from two ecologically distinct sites, one with permanent and the other with only temporary breeding habitats, during the rainy season and the transitional period into the dry season. Larvae were reared to adulthood under natural conditions and several traits were analysed including ovarian development, sub-cuticular fat body hypertrophy, body size, and energy reserves. Gonotrophic dissociation was significantly more frequent in *An. coluzzii* at the onset of the dry season, indicating a shift toward reproductive arrest. All three species exhibited increased body size and cuticular fat deposits during the transitional period, though with species-specific differences. Notably, only *An. coluzzii* showed significant increases in energy reserves (proteins, lipids, and carbohydrates) during the transition period. These adaptive responses differed between the study sites, suggesting the influence of breeding habitats. The findings highlight that species within the *An. gambiae* complex engage in distinct phenotypic trajectories at the onset of the dry season, suggesting divergent adaptations and trade-offs in energy acquisition and allocation to survive during the dry season.

## Abstract (fr)

Dans les savanes tropicales à longue saison sèche, les populations de moustiques vecteurs du paludisme disparaissent virtuellement après l’asséchement des gîtes larvaires, pour réapparaître en grand nombre au début de la saison des pluies suivante. Si l’estivation et les migrations sur de longues distances sont considérées comme des stratégies clés permettant à ces vecteurs de survivre durant la saison sèche, les caractéristiques physiologiques, biochimiques et morphologiques sous-jacentes à ces mécanismes restent insuffisamment étudiées, notamment en conditions naturelles. Cette étude explore les variations saisonnières d’*Anopheles coluzzii*, d’*An. gambiae* et d’*An. arabiensis* au début de la saison sèche dans les savanes arides du Burkina Faso, en Afrique de l’Ouest. Des larves en fin de stade larvaire ont été collectées sur deux sites écologiquement distincts, l’un présentant des gîtes larvaires permanents et l’autre uniquement des gîtes temporaires, durant la saison des pluies et la période de transition vers la saison sèche. Les larves ont été élevées jusqu’à l’âge adulte en conditions naturelles et plusieurs caractéristiques ont été analysées, notamment le développement ovarien, l’hypertrophie du corps gras sous-cuticulaire, la taille corporelle et les réserves énergétiques. La dissociation gonotrophique était significativement plus fréquente chez *An. coluzzii* au début de la saison sèche, indiquant un arrêt de la reproduction. Les trois espèces ont présenté une augmentation de leur taille corporelle et de leurs dépôts de graisse cuticulaire durant la période de transition, avec toutefois des différences spécifiques à chaque espèce. Notamment, seule *An. coluzzii* a montré une augmentation significative de ses réserves énergétiques (protéines, lipides et glucides) durant cette période. Ces réponses adaptatives différaient selon les sites d’étude, suggérant l’influence des habitats de reproduction. Ces résultats soulignent que les espèces du complexe *An. gambiae* adoptent des trajectoires phénotypiques distinctes au début de la saison sèche, suggérant des adaptations divergentes et des compromis dans l’acquisition et l’allocation d’énergie pour survivre durant cette période.

## Introduction

Despite intensive efforts to curb the disease, malaria remains a major global health burden. In 2023, 263 million human cases and 597,000 deaths were reported worldwide [81]. The majority of this burden is concentrated in African regions, where about 94% of cases and 90% of total deaths are reported. The primary malaria vectors in Africa are mosquitoes from the *Anopheles gambiae s.l.* complex and from the *Anopheles funestus* species group [76, 85]. *Anopheles coluzzii* Coetzee and Wilkerson 2013, (formerly M-form *An. gambiae*), *An. gambiae* Giles (formerly S-form *An. gambiae*), and *An. arabiensis* Patton are the most widespread and efficient vectors across Africa [9, 10]. These sibling species coexist in sympatry, but display key differences in traits such as host preference and larval ecology [9, 57, 84]. For instance, *An. gambiae* and *An. arabiensis* are typically associated with areas that have temporary breeding sites [11, 74], whereas *An. coluzzii* favours more stable, permanent larval habitats [4, 36].

In the West African savannah, the dry season can last for up to nine months, during which most standing water bodies and other surface water sources suitable for oviposition and larval development may completely dry up. This period is characterised by extreme environmental conditions with temperatures frequently exceeding 35 °C and relative humidity dropping below 10% [60, 61]. Ongoing climate change, with rising global temperatures, is likely to influence the distribution and (re-)emergence of vector-borne diseases such as malaria, West Nile fever, and dengue [29, 67, 71]. Understanding the mechanisms by which malaria vectors survive and persist through such a harsh dry season is crucial for guiding the development of alternative and more effective vector control strategies.

Several insects face harsh and variable environmental conditions and have thus evolved a wide range of survival mechanisms to cope with desiccation, heat stress, or food deprivation. These strategies include entering dormancy states such as diapause or quiescence [16, 17, 24, 56, 62, 79] and migration to areas with more favourable conditions [1, 8, 14, 33]. Several studies conducted on *Anopheles* mosquitoes [1, 15, 46–50, 56, 61, 65, 66, 75] support the hypothesis that population persistence during the dry season occurs primarily *via* aestivation in the form of adult females, particularly in *An. coluzzii,* as opposed to long-distance migration to and from areas with suitable breeding sites in *An. gambiae* and *An. arabiensis*. Under unfavourable conditions, the energetic cost of survival is generally higher, leading to potential trade-offs with other life-history components [72, 77]. As a consequence, organisms may initiate preparatory adjustments, including morphological and physiological adaptations in anticipation of these challenging conditions [2, 42–45, 54, 60]. Alterations in reproductive physiology, accumulation of fat reserves, changes in wing morphology, and reallocation of energy resources are among the most commonly documented traits associated with diapause [14, 18, 38, 59, 79].

Ovarian development is a physiological process by which female mosquitoes produce and mature eggs. It comprises two distinct phases (previtellogenic and vitellogenic stages), with ovarian development being triggered by the ingestion of a blood meal. In the absence of a blood meal, there is no progression beyond the pre-vitellogenic stage. When the ovaries or follicles of female mosquitoes that have taken a full blood meal fail to develop, the arrested ovarian development is termed gonotrophic dissociation [30, 82, 83].

During unfavourable periods, the fat body plays a vital role in energy storage and metabolic regulation, thus driving mosquito survival [3, 68]. Seasonal changes in fat body hypertrophy characterised by increased fat deposits and enlarged lipid droplets have been reported in numerous insects, including mosquitoes [3, 13, 41, 78]. In addition to the fat body, the accumulation of nutritional energy reserves, in particular glycogen and sugars [3], enables mosquitoes to withstand extended periods of environmental stress. As large body reserves increase survival probability, body size also serves as an indicator of adaptive responses in anticipation of diapause, with larger sizes often reflecting enhanced survival capacity [5]. Approaches such as geometric morphometrics, which assess shape, size, and allometric relationships, provide valuable insights into phenotypic variability under various environmental settings [6, 26, 28, 37].

In the present work, we investigated whether physiological and morphological changes occur in *An. coluzzii*, *An. gambiae*, and *An. arabiensis* in anticipation of the dry season, potentially as adaptive strategies to enhance survival during the prolonged and harsh environmental period. We hypothesize that i) the simultaneous occurrence of elevated temperatures, reduced relative humidity (RH), and increased larval crowding consequent to the progressive desiccation of aquatic breeding sites at the onset of the dry season, act as a set of token stimuli (predictive environmental cues) for mosquitoes, signalling upcoming inhospitable environmental conditions; ii) these stimuli trigger a cascade of physiological, behavioural, and morphological responses in mosquitoes, as a means for their adaptive survival strategies; and iii) these responses include gonotrophic dissociation, development of sub-cuticular fat deposits, increased teneral reserves, and larger body size, and may vary across species and populations sampled from different geographic locations. Beyond its ecological relevance, a better understanding of these seasonal survival mechanisms could support the development of novel malaria vector control interventions.

## Material and methods

### Study sites

**Figure S1:**
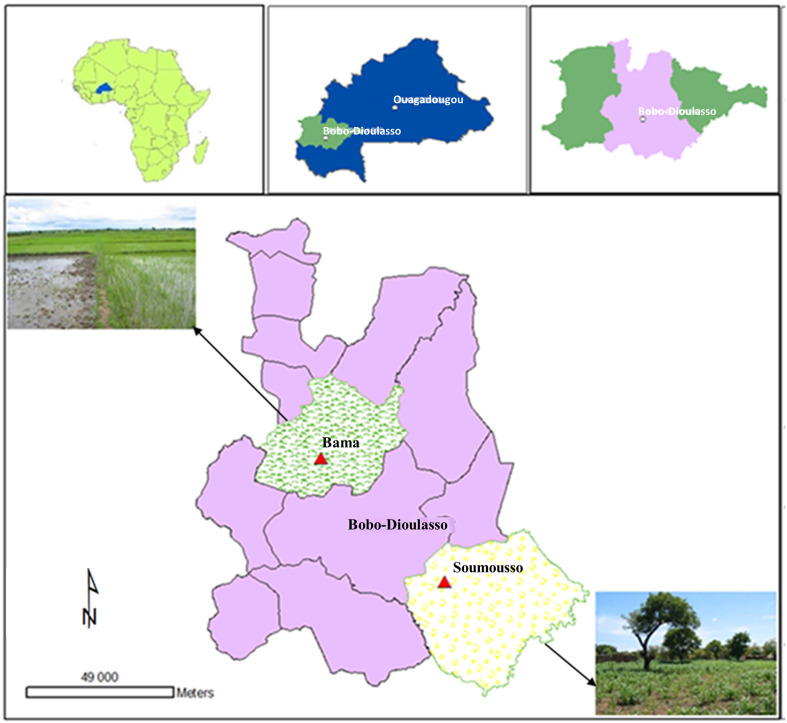
Geographic location of the two study sites in Burkina Faso (West Africa).

Two ecologically distinct sites, which differ in terms of mosquito population dynamics and the seasonal availability of breeding sites were selected for insect collections. The first site, Bama (11° 23′ N, 4° 24′ W), is a rice cultivation area situated approximately 30 km north of Bobo-Dioulasso (Southwestern Burkina Faso, West Africa) (Figure S1). The rice paddies are constantly irrigated with water from the nearby perennial Kou River, ensuring the presence of stable aquatic habitats throughout the year. These environmental conditions provide permanent habitats for mosquito breeding and are thus characterized by endemic malaria transmission [12, 35]. In this locality, *An. coluzzii* is the predominant malaria vector. The second site, Soumousso (11° 04′ N, 4° 03′ W), is a savannah area located 30 km east of Bobo-Dioulasso (Figure S1). In contrast to Bama, Soumousso experiences strong seasonal fluctuations in the availability of breeding sites, which directly influence vector abundance: *An. gambiae* and *An. arabiensis* are abundant in the rainy season when breeding sites are also abundant, but are very rarely found during the dry season due to the disappearance of breeding sites [20, 21, 70]. Following mosquito sampling from both study sites, larvae and pupae were transferred to Bama for transplantation into a large larval habitat present year-round and where a Vantage Pro2 weather station (Weatherlink; Davis Instruments, Hayward, CA, USA) was installed to continuously monitor environmental conditions. Temperature, relative humidity, and rainfall were recorded at hourly intervals.

### Experimental design

**Figure 1:**
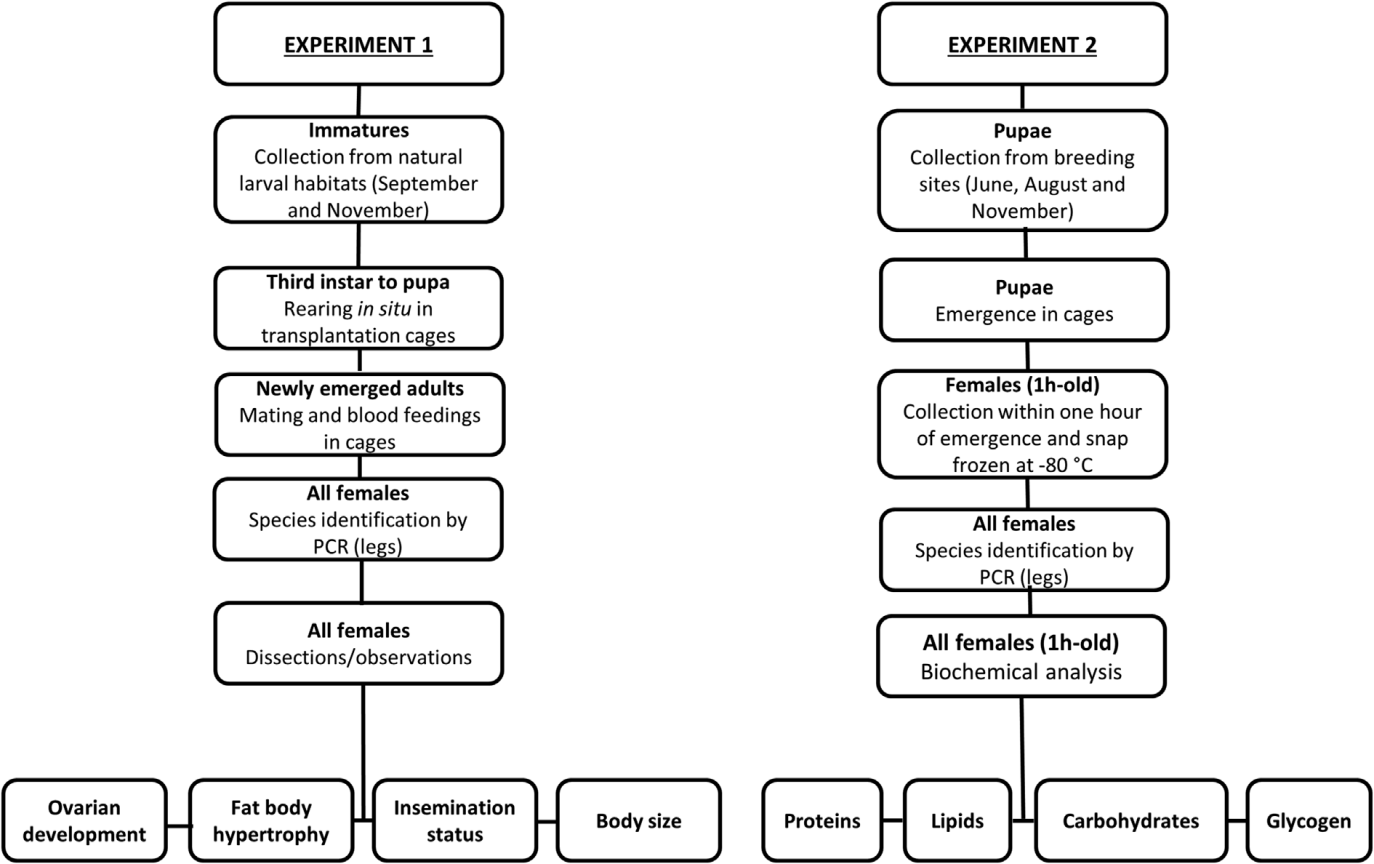
Experimental study design: Experiment 1 (left) and Experiment 2 (right).

The experimental design was divided into two distinct parts. The first part of the study (Experiment 1) focused on assessing ovarian development (gonotrophic dissociation), fat body hypertrophy (including sub-cuticular fat deposit and lipid droplets), insemination status, and geometric morphometrics (centroid size). The second part of the study (Experiment 2) was designed to investigate the dynamics of energy reserve allocation in adult females during the larval developmental stage. The overall experimental setup is illustrated in Figure 1.

### Experiment 1: Assessment of ovarian development, fat body hypertrophy, insemination status, and geometric morphometrics

The objective of this experiment was to investigate seasonal phenotypic variation in ovarian development, fat body hypertrophy, and centroid size.

#### Immature specimen sampling and transplantation

Immature mosquitoes were collected from natural larval habitats at the aforementioned study sites in 2011, during two key periods: (i) September, hereafter referred to as the rainy season, and (ii) November to December, hereafter referred to as the dry season. Aquatic habitats including small rain pools, cattle hoofprints, footprints, mud holes, and stagnant puddles near bridges or lowland rivers were surveyed for the presence of immature mosquitoes. Larvae were collected at both sites (Bama and Soumousso) using a 350 mL soup ladle and transferred to large holding containers. *Anopheline* larvae were visually sorted, and only late instar stages (third instar to pupa) were retained and counted. Late-instars were specifically selected based on the assumption that they had already been exposed to environmental token stimuli, thereby preconditioning the emerging adults to initiate survival-related physiological responses. To ensure natural larval development under identical biotic and climatic conditions until adult emergence, larvae and pupae from both study sites were transferred the same day and transplanted to a large natural breeding site located in Bama using transplant cages as described by Diabaté *et al.* [22, 23].

#### Adult emergence and blood feeding

Following adult emergence (between 1 to 3 days after larval transplantation), mosquitoes were collected in the early morning for several consecutive days using *Drosophila* tubes. Morphological identification was performed using standard taxonomic keys to confirm adults belonging to the *An. gambiae s.l* complex [34]. Identified adults were subsequently transferred to a large screened greenhouse enclosure (15 × 8 × 4.5 m) constructed with a metal frame and covered by mesh walls and a netted roof supported by metal structures, as described by Mamai *et al.* [61]. This setup effectively exposed mosquitoes to the prevailing environmental conditions. An equal number of male and female adult mosquitoes (50:50 sex ratio) were placed in a 30 × 30 × 30 cm cage to allow mating. A 10% glucose solution was provided *ad libitum* as a carbohydrate source. For blood feeding, restrained rabbits were positioned on top of the cage and served as a blood meal source for females on days 2, 4, 6, 8, and 10 post-emergence. Blood feeding occurred during the night from 07:30 to 08:30 pm, after which females were visually observed for the presence of blood in the abdomen.

Females lacking visible blood meals in their abdomens were removed and transferred to smaller cages for an additional one-hour feeding period. Females that successfully fed during either session were returned to the original cage, while individuals that failed to feed were excluded from the experiment. On the fourteenth day, mosquito cages were transported from the greenhouse to the laboratory of the Institut de Recherche en Sciences de la Santé (IRSS) in Bobo-Dioulasso, Burkina Faso. Upon arrival at the IRSS, females were promptly knocked down at −20 °C, after which they were individually isolated and stored at −80 °C for subsequent analysis.

#### Physiological and morphological assessments

Legs were carefully removed from each specimen and preserved in silica-gel desiccant for species identification using the SINE PCR method, as described by Santolamazza *et al.* [73]. The remaining carcasses were dissected under a microscope (Leica ICC 50) in a drop of physiological saline for the subsequent investigations. From a total of 39,016 late-stage immature mosquitoes collected, 1,946 blood-fed females were successfully identified across both study sites and subsequently included in the analysis.

**Figure 2:**
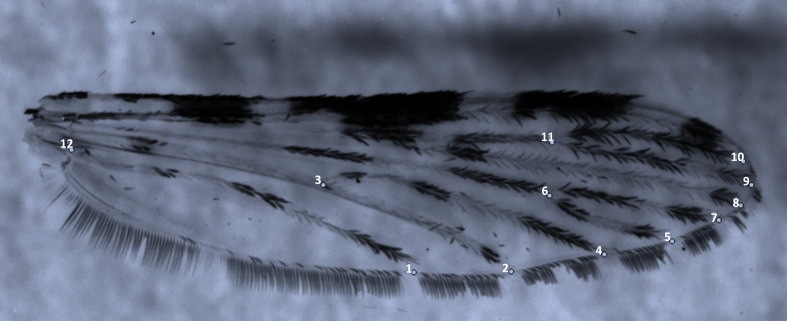
A left wing belonging to a member of the *Anopheles gambiae* species complex indicating the location of 12 landmarks used in geometric morphometrics analysis using CLIC software.

**Figure S2:**
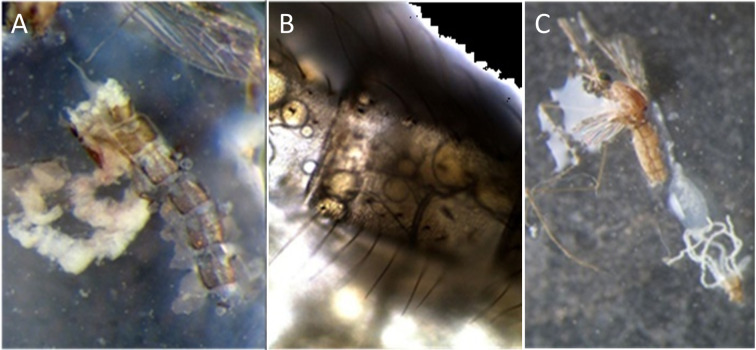
Phenotypes of subcuticular fat body hypertrophy in female *Anopheles gambiae* s.l., mosquitoes showing the presence of subcuticular fat deposits (A), lipid droplets (B), and absence of lipid reserves (C).

1) *Ovarian development* – ovaries were examined to determine the stage of follicular maturation, following the classification system established by Christophers and further refined by Detinova [19]. Ovarian stages 1 to 3 were observed using 100× and 200× magnification, while stages 4 and 5 were determined directly under the binocular microscope (40 × magnification);

2) *Fat body hypertrophy* (Figure S2) – the presence (Figure S2-A) or absence (Figure S2-C) of sub-cuticular fat deposits, along with associated lipid droplets (Figure S2-B) was assessed directly under a dissecting microscope;

3) *Insemination status* – a random subsample of 300 females, drawn from the total females collected at each site was analysed using the methodology previously reported in Tripet *et al.* [80]. The abdomen was excised and immersed in 70% ethanol for 5 days. Subsequently, the spermathecae was then extracted from the 8th segment and opened to determine the presence or absence of a sperm ball (which forms upon contact with ethanol);

4) *Geometric morphometrics* – the left wings of adult female mosquitoes were gently removed from the thorax using fine forceps, then dry-mounted onto microscope slides and covered with coverslips. The prepared slides were allowed to dry before imaging. Wing photographs were captured using a Leica binocular microscope equipped with a digital camera connected to a computer. The images were processed and analysed using standard geometric morphometric techniques. For each wing, 12 landmarks (vein crosses) were identified and digitised (Fig. 2) using the COO package of Collecting Landmarks for Characterization and Identification (CLIC) software [26]. Wing size was quantified using the isometric estimator known as “centroid size”, defined as the square root of the sum of the squared distances of each landmark from the centroid of the configuration [25]. Centroid size has been widely recognised as a robust and informative proxy of body size in insects [27, 52, 53].

### Experiment 2: Monitoring the dynamics of energy reserve allocation in newly emerged adult females

The goal of this experiment was to assess whether environmental conditions during larval development across distinct seasonal phases lead to differential allocation of energy reserves in newly emerged adult females (within the first hour of their adult life), prior to any post-emergence feeding or metabolic activity.

#### Immature specimen sampling

Immature specimens were collected at the aforementioned study sites from breeding habitats in 2011 during three distinct seasonal periods: (i) the onset of the rainy season (June), (ii) the peak of the rainy season (August), and (iii) the onset of the dry season (November). Only collected pupae were retained and placed in trays inside emergence cages (30 × 30 × 30 cm), which were checked regularly until emergence. Newly emerged female mosquitoes were collected at hourly intervals and immediately flash-frozen in liquid nitrogen to ensure minimal post-emergence metabolic transformation of larval-derived reserves. Upon return to the laboratory, the tubes containing the mosquitoes were stored at −80 °C for subsequent analysis of energy reserves.

#### Nutrient reserves

Legs were carefully removed from each specimen and preserved in silica-gel desiccant for species identification using the SINE PCR method [73]. Following successful molecular identification, 30 individuals per species, site, and collection period were used for nutrient quantification. From each mosquito specimen, the amount of body proteins, lipids, carbohydrates, and glycogen were quantified using colorimetry-based biochemical assays, following the protocol developed by Rivero *et al.* [69] and adapted by Mouline *et al.* [64].

Briefly, individual mosquitoes were homogenised in 70 μL of methanol, followed by the addition of 120 μL of methanol. The resulting homogenate was split into two equal aliquots of 70 μL each, one of which was used for protein quantification. To the second aliquot, 680 μL of chloroform: methanol (1:2) and 100 μL of sodium sulfate were added and homogenised. The final supernatant was divided equally for lipid and carbohydrate quantification, while the precipitate was used for glycogen determination. Protein concentrations were determined using the Bradford assay, and lipids using the vanillin reagent assay, while both carbohydrate and glycogen content were determined using an anthrone-based assay. Metabolite concentrations were calculated using standard curves specific to each biochemical compound.

### Statistical analysis

All statistical analyses were conducted using R software, version 4.3.2 (R Development Core Team 2008) along with the RStudio environment, version 2024.10.31. (RStudio, Inc. Boston, MA, USA, 2016). For ovarian development, females were pooled for pre-vitellogenic (*i.e.* Christophers’ stage I + II) and vitellogenic (III–V) stages, and Chi-square tests were used to compare their distribution across species, sites, and collection periods. Fisher’s exact tests were used to explore differences in the distribution of the three phenotypic classes of fat body hypertrophy across species, sites, and collection periods. Geometric morphometric analyses were based on landmark coordinate data, which were subjected to generalised procrustes superimposition and standard geometric morphometric procedures for multivariate analysis [26–28]. The centroide size was analysed using a Gaussian linear mixed-effects model, with season and species as fixed effects.

For nutrient reserves data analysis, one-way analysis of variance (ANOVA) followed by Tukey’s *post hoc* test was performed using GraphPad Prism, version 5.00. Graphical representations were produced using Microsoft Excel 2019. Normality and homoscedasticity of data were verified by Kolmogorov-Smirnov and Bartlett tests, respectively.

## Results

### Environmental conditions at the Bama study site

**Figure S3:**
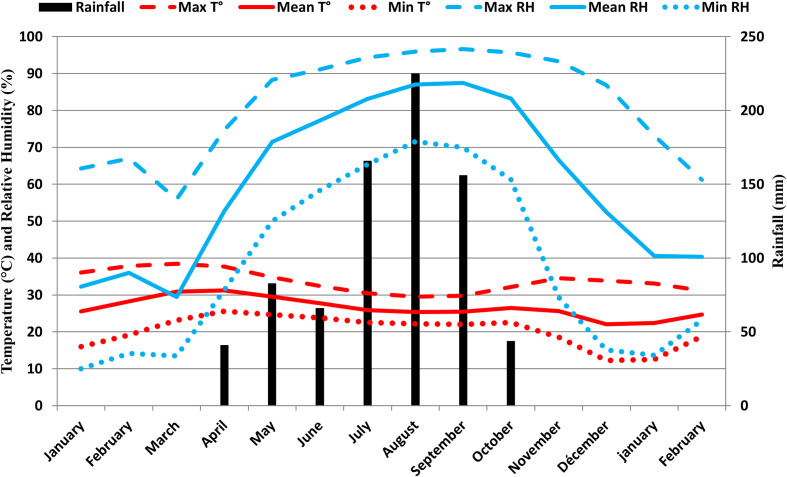
Climatic variation in temperature (°C), relative humidity (%), and total monthly rainfall (mm) variations at Bama (Burkina-Faso). Monthly maximum, mean, and minimum temperatures (T°, red lines), relative humidity (RH, blue lines), and rainfall (black vertical bars).

Climatic variables, including monthly average temperatures (°C), relative humidity (%), and total yearly rainfall (mm), measured at the study site in Bama are presented in Figure S3. As expected, the area exhibited pronounced fluctuations in temperature and humidity regimes, allowing for the definition of three distinct phases:

- Rainy season (from mid-June to mid-October). This phase accounts for the majority of annual precipitation (about 740 mm) and is characterized by moderately low and stable temperatures (*e.g.* range 22–30 °C), along with high (>50%) relative humidity levels;
- Transitional period (November–December). This phase marks the onset of the dry season and is characterized by substantial diurnal variations, with extreme values ​​for both temperature (*e.g.* range 12–35 °C) and relative humidity (10–93%). The broad thermal amplitude is attributed to marked night-time cooling, with temperatures occasionally dropping to as low as 10 °C;
- Hot dry season (January to mid-June). This period is characterised by extreme daytime temperatures (up to 38.4 °C), persistently low relative humidity (~10%), and a narrower diurnal thermal range compared to the transitional phase, largely due to elevated night-time temperatures. Notably, during the transitional period between rainy and dry seasons (*i.e.* onset of dry season), most larval breeding sites dried up at our study sites. This phase potentially corresponds to the preparatory stage of aestivation in *Anopheles* mosquitoes, during which key physiological adaptations for survival are likely initiated.

### Species composition

A total of 39,016 late-stage immature specimens (L3 to pupa) belonging to *An. gambiae* complex were collected across the two study sites in Experiment 1. PCR analysis successfully identified 1,946 females 14 days after successful blood feeding. Species-specific distribution by location revealed that only *An. coluzzii* was found in Bama, accounting for 100% (*n* = 803) of the females identified at this site. In contrast, among the 1,143 specimens identified in Soumousso, *An. gambiae* was the most abundant, representing 48.47% (*n* = 554), followed by *An. arabiensis* (29.66%, *n* = 339), and *An. coluzzii* (21.87%, *n* = 250).

### Ovarian development

Dissection of the spermathecae from 300 females from both sites revealed an insemination rate of 98%, indicating that the observed ovarian development reflects normal reproductive biology and is not attributable to insemination failure.

**Figure 3:**
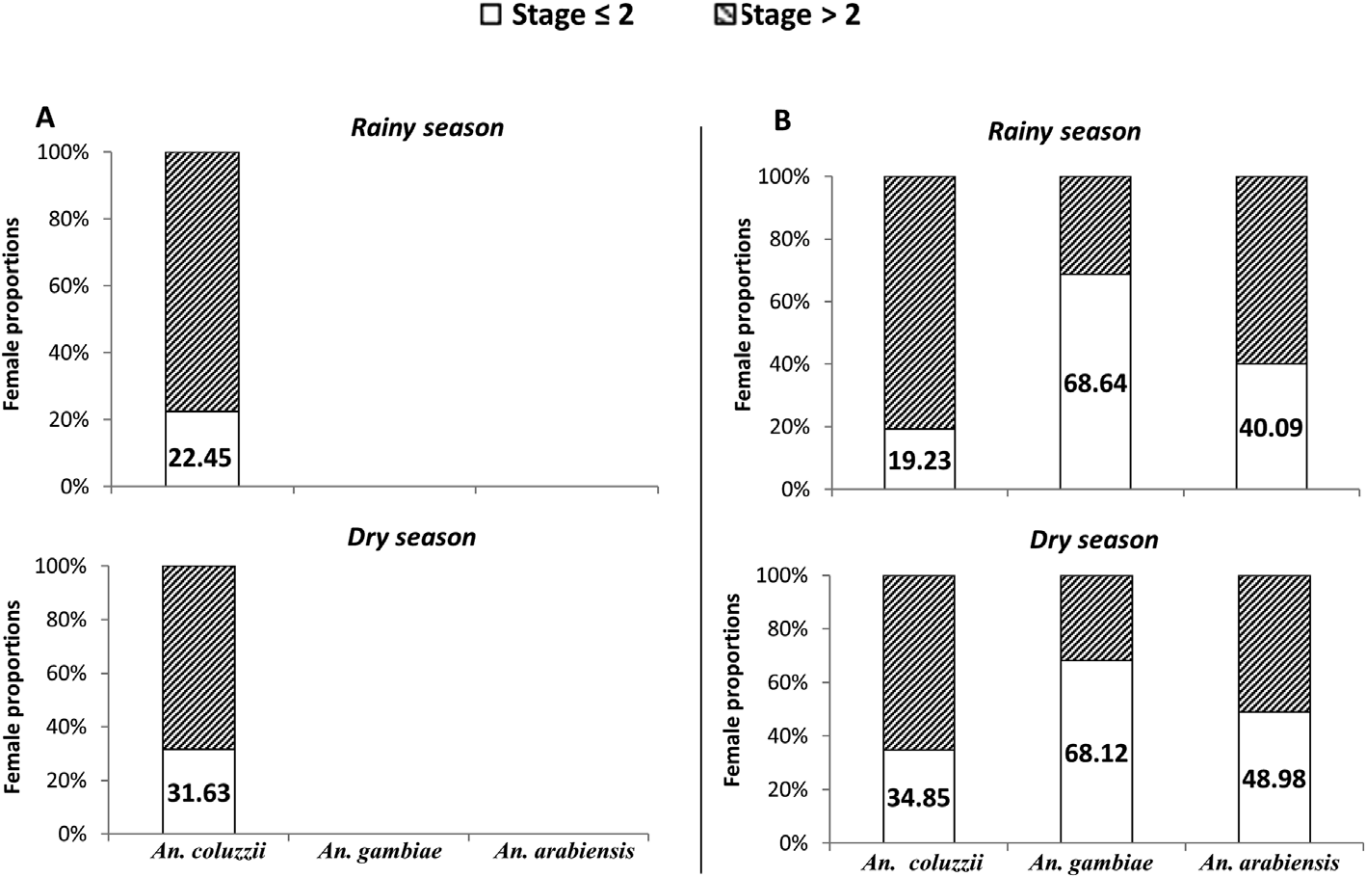
Ovarian developmental stages in female *Anopheles coluzzii*, *Anopheles gambiae*, and *Anopheles arabiensis* during the rainy and at the onset of the dry seasons, and across two study sites in Burkina Faso. A = Bama (permanent breeding sites); B = Soumousso (temporary breeding sites).

Regardless of the season, *An. coluzzii* was the species that most efficiently developed eggs beyond ovarian stage 2 (ranging from 65.15 to 80.77%) compared to both *An. arabiensis* (51.02–59.91%) and *An. gambiae* (31.36–31.88%) (Fig. 3, Table 1). Despite multiple blood meals being provided, 19.23 to 68.64% of female mosquitoes collected at both localities failed to develop their ovaries beyond Christopher’s stage 2, indicating a physiological state of gonotrophic dissociation. Interestingly, *An. coluzzii* from both sites showed significantly higher proportions of females in gonotrophic dissociation at the onset of the dry season than during the rainy season (χ^2^ = 3.953, df = 1, *p* = 0.004 and χ^2^ = 5.584, df = 1, *p* = 0.018 for Soumousso and Bama populations, respectively). By contrast, no significant seasonal variation in the proportion of females in gonotrophic dissociation was evidenced in *An. gambiae* (χ^2^ = 0.00066, df = 1, *p* = 0.979) and *An. arabiensis* females (χ^2^ = 1.846, df = 1, *p* = 0.17) from Soumousso (Table 1).

**Table 1:** Statistical analyses comparing the effects of season, locality, and species on phenotypic traits in *Anopheles* mosquitoes. Results present probabilities from Chi-square (χ^2^) tests for gonotrophic dissociation, Fisher’s exact tests for fat body hypertrophy (lipid droplets and fat deposits), and linear mixed model (LMM) analyses for wing centroid size. Significant differences (*p* < 0.05) indicate the influence of season (rainy *vs.* dry), study locality (Bama *vs.* Soumousso), and interspecific variation (*Anopheles coluzzii, Anopheles gambiae*, and *Anopheles arabiensis*) on these traits.

Parameters	Type of comparison Species and/or locality		*p*-value
Gonotrophic dissociation	Rainy season *vs* dry season	*An. coluzzii (Bama)*	**0.018**
		*An. coluzzii (Soumousso)*	**0.046**
		*An. gambiae (Soumousso)*	0.979
		*An. arabiensis (Soumousso)*	0.174
	Permanent site *vs* temporary site	*An. coluzzii*	0.452
	*An. coluzzii* vs *An. gambiae Soumousso*		**4.156e-11**
	*An. coluzzii* vs *An. arabiensis Soumousso*		**0.026**
	*An. gambiae* vs *An. arabiensis Soumousso*		**0.002**
	
Lipid droplets	Rainy season *vs* dry season	*An. coluzzii (Bama)*	**< 2.2e-16**
		*An. coluzzii (Soumousso)*	**0.002**
		*An. gambiae (Soumousso)*	**0.028**
		*An. arabiensis (Soumousso)*	0.112
	Permanent site *vs* temporary site	*An. coluzzii*	**8.963e-05**
	*An. coluzzii* vs *An. gambiae Soumousso*		**2.288e-10**
	*An. coluzzii* vs *An. arabiensis Soumousso*		1
	*An. gambiae* vs *An. arabiensis Soumousso*		**0.027**
		
Fat deposits	Rainy season *vs* dry season	*An. coluzzii (Bama)*	**0.0001**
		*An. coluzzii (Soumousso)*	**1.412e-06**
		*An. gambiae (Soumousso)*	**< 2.2e-16**
		*An. arabiensis (Soumousso)*	**< 2.2e-16**
		
	Permanent site *vs* temporary site	*An. coluzzii*	**9.337e-15**
	*An. coluzzii* vs *An. gambiae Soumousso*		**2.816e-12**
	*An. coluzzii* vs *An. arabiensis Soumousso*		**0.003**
	*An. gambiae* vs *An. arabiensis Soumousso*		**0.007**
Adult centroid size	Rainy season *vs* dry season	*An. coluzzii*	**3e-04**
		*An. gambiae*	0.312
		*An. arabiensis*	0.441
	*An. coluzzii* vs *An. gambiae Soumousso*		0.092
	*An. coluzzii* vs *An. arabiensis Soumousso*		0.129
	*An. gambiae* vs *An. arabiensis Soumousso*		**0.001**

### Fat body hypertrophy

**Figure 4:**
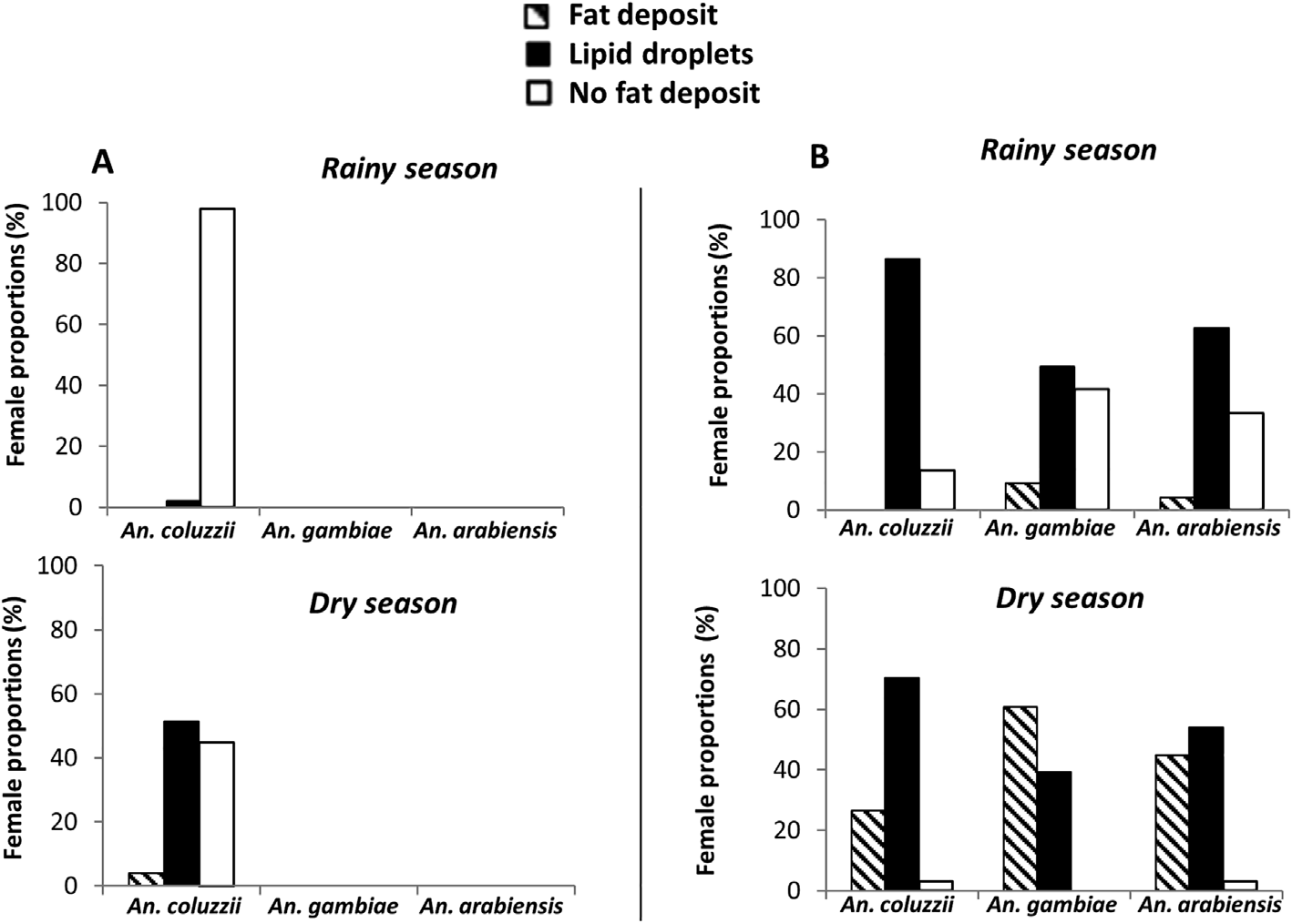
Proportion of *Anopheles coluzzii*, *Anopheles gambiae*, and *Anopheles arabiensis* females with sub-cuticular fat body hypertrophy collected from two different sites of Burkina Faso. A = permanent (Bama); B = temporary (Soumousso). See Supplementary Material S2 for phenotype description.

The proportion of females with subcuticular fat body hypertrophy in the form of fat deposit was significantly higher at the onset of the dry season by comparison to the rainy season across the three *Anopheles* species, and at both sites for *An. coluzzii* (*p* < 0.05, Fig. 4, Table 1), although this trend was more marked in Soumousso (temporary) than in Bama (permanent) (*p* < 0.05). In turn, the proportion of females with no evidence for fat body hypertrophy (*i.e.* no fat deposit) was higher during the rainy than at the onset of the dry season in all species, accounting for over 98% of adult *An. coluzzii* females collected in Bama during the rainy season. At the onset of the dry season, very few female mosquitoes were deprived of lipid reserves in Soumousso, regardless of the species, whereas in Bama, they still represented over 40% of *An. coluzzii* females. The proportion of females showing subcuticular lipid droplets was highest in Soumousso during the rainy season, in all three species, although with statistically significant differences in their distribution between species. It was also the major phenotypic class observed at the onset of the dry season in *An. arabiensis*, as well as in *An. coluzzii* females from both sites. The proportion of females with lipid droplets was significantly higher at the onset of the dry season than in the rainy season in *An. coluzzii* in Bama (*p* < 0.05, Table 1), whereas *An. arabiensis* showed no seasonal difference (*p* = 0.112). Within *An. coluzzii*, the proportion of females with lipid droplets was higher at the temporary site (Soumousso) than at the permanent site (Bama), at both time points (*p* < 0.05, Table 1).

Interestingly, at the onset of the dry season in Soumousso, the proportion of females of all three species in a state of gonotrophic dissociation were significantly more likely to possess subcuticular fat deposits and lipid droplets (Fisher test, *p* < 0.05).

### Adult body size

**Figure 5:**
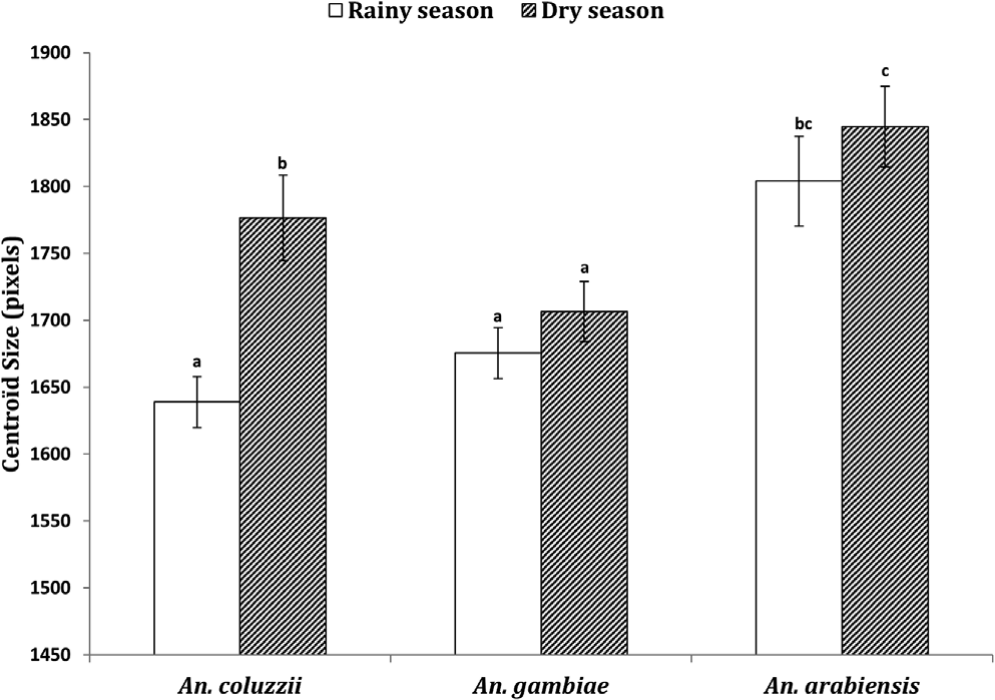
Wing centroid size in *Anopheles coluzzii*, *Anopheles gambiae*, and *Anopheles arabiensis* collected during the rainy season and at the onset of the dry season at two sites in Burkina Faso*.* Different letters indicate statistically different *p*-values.

The isometric estimator of the adult body size, measured as wing centroid size*,* is shown in Figure 5. A total of 107 individuals were analysed: *An. coluzzii* (*n* = 47), *An. gambiae* (*n* = 32), and *An. arabiensis* (*n* = 28). In particular, *An. coluzzii* exhibited significantly larger body size at the onset of the dry season (LMM, df = 45, *t* = 3.914, *p* = 3e-04) compared to the rainy season. However, this difference was not significant in *An. gambiae* (LMM, df = 30, *t* = 1.028, *p* = 0.312) and *An. arabiensis* (LMM, df = 26, *t* = 0.783, *p* = 0.441). Across all seasons, *An. arabiensis* females were significantly larger than *An. coluzzii* (LMM, df = 73, *t* = −4.301, *p* = 1e–04) and *An. gambiae* females (LMM, df = 58, *t* = −5.199, *p* < 0.005).

### Nutrient reserves

**Figure 6:**
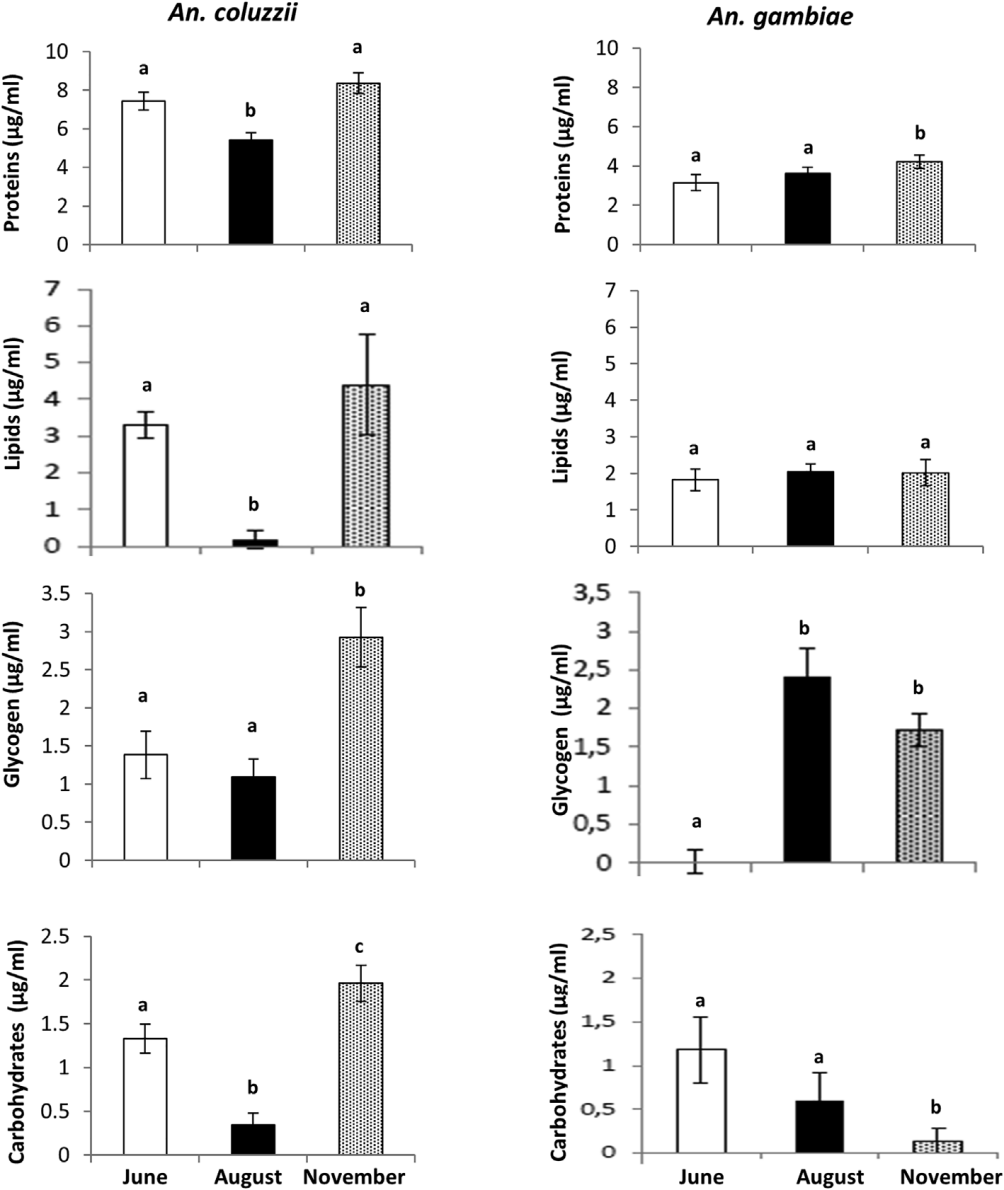
Nutritional reserves (mean ± SE) in newly emerged *Anopheles coluzzii* and *Anopheles gambiae.* Different letters indicate significant differences in means (*t*-test, *p* < 0.05).

At each study site, only one species was found (collected) in sufficient numbers for analysis, *i.e. An. coluzzii* from Bama (permanent breeding sites) and *An. gambiae* from Soumousso. As a result, analyses were unfortunately restricted to these two mosquito species. The results presented in Figure 6 revealed significant seasonal variation in nutrient reserves (ANOVA, F = 28.39, df = 11, *p* < 0.001 and *F* = 19.17, df = 11, *p* < 0.001, respectively for *An. coluzzii* and *An. gambiae).* In *An. coluzzii,* protein, lipid, and carbohydrate contents were significantly lower during the peak of the rainy season (August) compared to the onset of the rainy season (June) (Fig. 6, Tukey’s *post hoc* test, *p* < 0.005), while glycogen levels remained stable. At the onset of the dry season (November), protein, lipid, and carbohydrate reserves significantly increased relative to the levels observed in the peak of the rainy season (August) (Tukey’s *post hoc* test, *p* < 0.005). In contrast, *An. gambiae* exhibited no significant changes in protein, lipid, and carbohydrate contents between the onset (June) and the peak (August) of the rainy season (Tukey’s *post hoc* test, *p* > 0.005)*.* At the onset of the dry season, lipid and glycogen levels remained unchanged, whereas carbohydrate levels decreased and protein levels increased significantly (Tukey’s *post hoc* test, *p* < 0.005). Overall, *An. coluzzii* accumulated higher nutrient reserves than *An. gambiae*, both at the onset of the rainy season and at the onset of the dry season (Tukey’s *post hoc* test, *p* < 0.005).

## Discussion

Previous and recent studies have provided compelling evidence that certain members of the *An. gambiae* s.l. complex can persist over the duration of the dry season in tropical savannahs through mechanisms such as aestivation or long-distance migration [1, 15, 56, 61, 63]. However, the physiological, biochemical, and morphological traits underpinning these survival strategies remain insufficiently explored, particularly under natural field conditions. In this study, we investigated physiological (gonotrophic dissociation, fat-body hypertrophy), biochemical (level of glycogen, sugars, proteins, and lipids), and morphological (body size) traits in mosquito populations from two ecologically distinct sites in Bobo-Dioulasso (Burkina Faso, West Africa), during the rainy season and at the onset of the dry season, with the aim of identifying signatures of adaptations to the harsh period.

*Anopheles coluzzii* females collected from Soumousso exhibited a significantly higher incidence of gonotrophic dissociation at the onset of the dry season compared to the rainy season. In these females, ovaries remained in the pre-vitellogenic stage of development (up to stage 2 according to Christopher’s classification) despite the ingestion of multiple blood meals, indicating a state of reproductive arrest. These findings are consistent with previous studies reported by Yaro *et al.* [86], who documented gonotrophic dissociation in *An. coluzzii* collected indoors during the dry season in an arid region of Mali. In that study, reproductive arrest was not limited to arrested ovarian development, but also involved suppressed oviposition, even in females with fully developed eggs. Similar trends of gonotrophic dissociation had previously been reported in *An. arabiensis* in the Sudan [65], in hibernating females of *An. earlei* [32] and in *An. punctipennis* [58], suggesting broader ecological relevance of this strategy among *Anopheles* species facing unfavourable environmental conditions.

The results of this study did not show significant seasonal variation in gonotrophic dissociation in either *An. gambiae* and *An. arabiensis*. Moreover, a surprisingly high proportion of females from both species exhibited gonotrophic discordance even during the rainy season, when environmental conditions are generally most favourable for reproduction. This has been observed in *An. gambiae* under insectary conditions, where reduced ovarian maturation was noted compared to *An. coluzzii* [64]. *Anopheles gambiae* and *An. arabiensis*, which typically colonise temporary habitats, may have evolved more plastic reproductive responses than specific dry-season survival strategies. This plasticity is likely shaped by a combination of complex factors, including carryover effects from larval environments such as intense competition for food, exposure to sublethal stressors, or predation pressure. Additionally, certain genotypes within these populations may be inherently more prone to exhibit gonotrophic discordance. The assumption that *An. gambiae* might be more physiologically sensitive to environmental stressors than *An. coluzzii* and *An. arabiensis* cannot be excluded, especially given the well-documented difficulties in establishing stable insectary colonies of the latter species. However, these hypotheses deserve further investigation.

Fat body hypertrophy (characterised by a subcuticular fat layer) and the presence of lipid droplets were observed in females of the three species, with the highest prevalence in *An. gambiae*. In holometabolous insects, fat body serves as a key metabolic organ, functioning as the principal site for the storage of lipids, glycogen, and protein. Lipid accumulation leads to hypertrophy of the fat body, a condition known to enhance survival in hibernating *Culex pipiens (Diptera: Culicidae)* females [3]. Moreover, lipids represent the primary energy reserves utilised during insect diapause [41]. In *Anopheles messeae,* fat body formation during hibernation has been attributed to nutrients derived from either larval feeding or adult blood meals [51]. The accumulation of such reserves is recognised as a prerequisite for diapause induction in many insects [13, 41]. Ultimately, the survival of an individual during adverse conditions, such as the extended dry season, depends largely on its capacity to acquire and store sufficient energy to compensate for resource deficits. Supporting this, lipid accumulation has been reported in *Drosophila* under nutrient stress [7] and in *An. gambiae* under desiccation stress [40].

Interestingly, our study also revealed phenotypic variation between the two selected study sites. Although the two localities display similar trends in climatic conditions, they differ markedly in the temporal availability of aquatic habitats. Soumousso represents a typical wet savannah environment, where breeding sites are exclusively rainfall-dependent, resulting in vector breeding activity that closely follows the rainy season. In contrast, Bama is characterised by irrigated rice cultivation, which ensures the presence of permanent breeding sites throughout the year, allowing for continuous mosquito proliferation. The phenotypic variation observed in *An. coluzzii* between temporary *vs.* permanent breeding sites likely reflects its remarkable adaptability to anthropogenic environments and underscores its ecological plasticity. The capacity to colonise permanent water bodies may enable *An. coluzzii* to maintain reproductive activity even during the dry season. Thus, in areas where permanent breeding sites are present year-round, this species could invest either in survival by accumulating nutrient reserves or continued reproduction. Conversely, in areas where oviposition sites disappear during the dry season, a significant proportion of individuals may shift their physiology towards a state of gonotrophic discordance, as suggested by our results.

Newly emerged *An. coluzzii* accumulated significantly higher levels of lipids, sugars, glycogen, and protein during the transitional period (*i.e.* at the onset of the dry season) compared to the rainy season. This substantial accumulation of energy reserve likely reflects the enhanced ability of this species to cope with desiccation stress, thereby promoting greater adult survival, as previously demonstrated by Lee *et al.* [55]. Such physiological conditioning may contribute to its continuous presence in Bama throughout the year [4, 36] and also suggests a potential capacity for aestivation in response to the temporary nature of breeding habitats in Soumousso [56]. However, it should be noted that nutrient reserves in experiment 2 were expressed in mg/mL without standardisation to mosquito body size or weight, which represents a limitation of this study. Consequently, variations between seasons or species may partly reflect differences in mosquito size rather than true physiological differences. Future studies should include size- or mass adjusted biochemical quantification to more accurately assess seasonal or species-specific metabolic strategies.

As expected in our study, females collected at the onset of the dry season exhibited significantly larger body sizes than those collected during the rainy season, particularly in *An. coluzzii*. Body size was assessed using centroid size, a geometric morphometric measure considered to be a more informative and efficient estimator of body size compared to traditional metrics, such as wing length [52, 53]. The higher body size observed in *An. coluzzii* supports the general hypothesis that under adverse environments, natural selection would favour larger phenotypes. An alternative explanation for the observed size variation is diapause preparation for aestivation. Benoit and Denlinger [5] indeed demonstrated that diapausing individuals tend to exhibit greater body sizes compared to their reproductively active counterparts. Additionally, it is known that body size at emergence is closely linked to the quality of larval nutrition, and larger individuals are more resistant to desiccation [31, 39]. Therefore, the increased body size observed in *An. coluzzii* at the onset of the dry season may indicate a physiological adaptation involving the accumulation of substantial energy reserves in anticipation of the dry period.

Based on these results, we cannot definitively separate the assumptions of aestivation and long-distance migration as alternative survival strategies employed by different *Anopheles* species. Phenotypes such as fat body hypertrophy, documented here to the best of our knowledge for the first time in *Anopheles*, and gonotrophic dissociation, while commonly associated with aestivation/diapause, are not exclusive indicators of this state and may also occur in individuals preparing for migratory behaviour. It is plausible that the accumulated lipid reserves serve as an energy source for sustained flight, while the observed subcuticular fat deposition in all three species may reflect preparatory physiological adaptations for migration toward more favourable habitats. Future studies integrating ecological tracking methods such as mark-release-recapture, with the identification of molecular or genetic biomarkers, would enhance our understanding of aestivation and migratory behaviours in *An. gambiae* s.l.

## Conclusion

Several physiological, biochemical, and morphological changes are triggered in *An. gambiae* s.l. mosquitoes at the onset of the dry season in the tropical savannahs of Burkina Faso, West Africa. The observed inter- and intra-specific variability in the expression of these traits likely reflects differential trade-offs in resources acquisition, allocation and utilisation, enabling populations to cope with environmental stressors and ecological constraints associated with seasonal changes. Unravelling the biological mechanisms underpinning the population dynamics of major human malaria vectors in tropical Africa is key to sustainable disease prevention and control, especially in a context of climate change and global warming.

## Data Availability

All data generated or analysed during this study are included in this published article.

## Supplementary material

The supplementary material of this article is available at https://www.parasite-journal.org/10.1051/parasite/2026010/olm.

## References

[1] Adamou A, Dao A, Timbine S, Kassogué Y, Yaro AS, Diallo M, Traoré SF, Huestis DL, Lehmann T. 2011. The contribution of aestivating mosquitoes to the persistence of *Anopheles gambiae* in the Sahel. Malaria Journal, 10, 151. 21645385 10.1186/1475-2875-10-151 PMC3123247

[2] Arcaz AC, Huestis DL, Dao A, Yaro AS, Diallo M, Andersen J, Blomquist GJ, Lehmann T. 2016. Desiccation tolerance in *Anopheles coluzzii*: the effects of spiracle size and cuticular hydrocarbons, Journal of Experimental Biology, 219, 1675–1688. 27207644 10.1242/jeb.135665 PMC4920233

[3] Arrese EL, Soulages JL. 2010. Insect fat body: energy, metabolism, and regulation. Annual Review of Entomology, 55, 207–225. 10.1146/annurev-ento-112408-085356 PMC3075550 19725772

[4] Baldet T, Diabaté A, Guiguemdé T. 2003. Étude de la transmission du paludisme en 1999 dans la zone rizicole de la vallée du Kou (Bama), Burkina Faso. Cahiers de Santé, 13, 55–60. 12925325

[5] Benoit JB, Denlinger DL. 2007. Suppression of water loss during adult diapause in the northern house mosquito, *Culex pipiens**.* Journal of Experimental Biology, 210, 217–226. 17210959 10.1242/jeb.02630

[6] Camara M, Caro-Riaño H, Ravel S, Dujardin J-P, Hervouet J-P, De Meeüs T, Kagbadouno MS, Bouyer J, Solano P. 2006. Genetic and morphometric evidence for population isolation of *Glossina palpalis gambiensis* (Diptera: Glossinidae) on the Loos islands, Guinea. Journal of Medical Entomology, 43, 853–860. 17017219 10.1603/0022-2585(2006)43[853:gamefp]2.0.co;2

[7] Chippindale AK, Chu TJF, Rose MR. 1996. Complex trade-offs and the evolution of starvation resistance in *Drosophila melanogaster**.* Evolution, 50, 753–766. 28568920 10.1111/j.1558-5646.1996.tb03885.x

[8] Clark MS, Worland MR. 2008. How insects survive the cold: molecular mechanisms-a review. Journal of Comparative Physiology. B, Biochemical, Systemic, and Environmental Physiology, 178, 917–933. 18584182 10.1007/s00360-008-0286-4

[9] Coetzee M, Craig M, Sueur D Le. 2000. Distribution of African malaria mosquitoes belonging to the *Anopheles gambiae* complex. Parasitology Today, 16, 74–77. 10652493 10.1016/s0169-4758(99)01563-x

[10] Coetzee M, Hunt R, Wilkerson R, Della TA, Coulibaly M, Besansky N. 2013. *Anopheles coluzzii* and *Anopheles amharicus*, new members of the *Anopheles gambiae* complex. Zootaxa, 3619, 246–274. 26131476

[11] Costantini C, Ayala D, Guelbeogo WM, Pombi M, Some CY, Bassole IH, Ose K, Fotsing J-M, Sagnon N, Fontenille D, Besansky NJ, Simard F. 2009. Living at the edge: biogeographic patterns of habitat segregation conform to speciation by niche expansion in *Anopheles gambiae**.* BMC Ecology, 9, 16. 19460144 10.1186/1472-6785-9-16 PMC2702294

[12] Dabiré KR, Diabaté A, Paré-Toé L, Rouamba J, Ouari A, Fontenille D, Baldet T. 2008. Year to year and seasonal variations in vector bionomics and malaria transmission in a humid savannah village in west Burkina Faso. Journal of the Society for Vector Ecology, 33, 70–75. 10.3376/1081-1710(2008)33[70:ytyasv]2.0.co;2 18697309

[13] Danks HV. 2006. Key themes in the study of seasonal adaptations in insects II. Life-cycle patterns. Applied Entomology and Zoology, 41, 1–13.

[14] Danks H. 1987. Insect dormancy: an ecological perspective. Ottawa: Biological Survey of Canada (Terrestrial Arthropods).

[15] Dao A, Yaro AS, Diallo M, Timbiné S, Huestis DL, Kassogué Y, Traoré AI, Sanogo ZL, Samaké D, Lehmann T. 2014. Signatures of aestivation and migration in Sahelian malaria mosquito populations. Nature, 516, 387–390. 25470038 10.1038/nature13987 PMC4306333

[16] Denlinger DL. 1986. Dormancy in tropical insects. Annual Review of Entomology, 31, 239–264. 10.1146/annurev.en.31.010186.001323 3510585

[17] Denlinger D. 2002. Regulation of diapause. Annual Review of Entomology, 47, 93–122. 10.1146/annurev.ento.47.091201.145137 11729070

[18] Denlinger D, Yocum G, Rinehart J. 2012. Hormonal control of diapause. Insect Endocrinology, 1, 430–463.

[19] Detinova T. 1962. Age grouping methods in diptera of medical importance with special reference to some vectors of malaria. Series No. 47. Geneva: World Health Organization Monograph Series. 13885800

[20] Diabate A, Baldet T, Chandre F, Akoobeto M, Guiguemde TR, Darriet F, Brengues C, Guillet P, Hemingway J, Small GJ, Hougard JM. 2002. The role of agricultural use of insecticides in resistance to pyrethroids in *Anopheles gambiae* s.l. in Burkina Faso. American Journal of Tropical Medicine and Hygiene, 67, 617–622. 12518852 10.4269/ajtmh.2002.67.617

[21] Diabate A, Brengues C, Baldet T, Dabiré KR, Hougard JM, Akogbeto M, Kengne P, Simard F, Guillet P, Hemingway J, Chandre F. 2004. The spread of the Leu-Phe kdr mutation through *Anopheles gambiae* complex in Burkina Faso: genetic introgression and *de novo* phenomena. Tropical Medicine & International Health, 9, 1267–1273. 15598258 10.1111/j.1365-3156.2004.01336.x

[22] Diabaté A, Dabiré RK, Heidenberger K, Crawford J, Lamp WO, Culler LE, Lehmann T. 2008. Evidence for divergent selection between the molecular forms of *Anopheles gambiae*: role of predation. BMC Evolutionary Biology, 8, 5. 18190719 10.1186/1471-2148-8-5 PMC2217532

[23] Diabaté A, Dabire RK, Kim EH, Dalton R, Millogo N, Baldet T, Simard F, Gimnig JE, Hawley WA, Lehmann T. 2005. Larval development of the molecular forms of *Anopheles gambiae* (Diptera: Culicidae) in different habitats: a transplantation experiment. Journal of Medical Entomology, 42, 548–553. 16119542 10.1093/jmedent/42.4.548

[24] Diniz DFA, de Albuquerque CMR, Oliva LO, de Melo-Santos MAV, Ayres CFJ. 2017. Diapause and quiescence: dormancy mechanisms that contribute to the geographical expansion of mosquitoes and their evolutionary success. Parasites & Vectors, 10, 310. 28651558 10.1186/s13071-017-2235-0 PMC5485599

[25] Dryden IL, Mardia KV. 2016. Statistical shape analysis: with applications in R (Second edition). United Kingdom: John Wiley & Sons.

[26] Dujardin J-PAL, Kaba D, Henry AB. 2010. The exchangeability of shape. BMC Research Notes, 3, 266. 20964872 10.1186/1756-0500-3-266 PMC2987866

[27] Dujardin J-P. 2008. Morphometrics applied to medical entomology. Infection, Genetics and Evolution, 8, 875–890. 10.1016/j.meegid.2008.07.011 18832048

[28] Dujardin JP. 2011. Modern morphometrics of medically important insects. Genetics and Evolution of Infectious Diseases, 1, 473–501.

[29] Ebi KL. 2014. Health in the new scenarios for climate change research. International Journal of Environmental Research and Public Health, 11, 30–46. 10.3390/ijerph110100030 PMC3924435 24452253

[30] Eldridge B. 1966. Environmental control of ovarian development in mosquitoes of the *Culex pipiens* complex. Science, 151, 826–830. 17746730 10.1126/science.151.3712.826

[31] Fouet C, Gray E, Besansky NJ, Costantini C. 2012. Adaptation to aridity in the malaria mosquito *Anopheles gambiae*: Chromosomal inversion polymorphism and body size influence resistance to desiccation. PloS One, 7, e34841. 22514674 10.1371/journal.pone.0034841 PMC3325948

[32] Gallaway WJ, Brust RA. 1982. Blood-feeding and gonotrophic dissociation in overwintering *Anopheles earli* (Diptera: Culicidae) from southern Manitoba. Canadian Entomologist, 114, 1105–1107.

[33] Gatehouse A. 1997. Behavior and ecological genetics of wind-borne migration by insects. Annual Review of Entomology, 42, 475–502. 10.1146/annurev.ento.42.1.475 15012321

[34] Gillies MT, Coetzee M. 1987. *Anophelines* mosquitoes: A supplement to the *Anophelinae* of Africa south of the Sahara (Afrotropical region). South African Institute for Medical Research, 55, 143.

[35] Gimonneau G, Bouyer J, Morand S, Besansky NJ, Diabate A, Simard F. 2010. A behavioral mechanism underlying ecological divergence in the malaria mosquito *Anopheles gambiae**.* Behavioral Ecology, 21, 1087–1092. 22476108 10.1093/beheco/arq114 PMC2920295

[36] Gimonneau G, Pombi M, Choisy M, Morand S, Dabiré RK, Simard F. 2012. Larval habitat segregation between the molecular forms of the mosquito *Anopheles gambiae* in a rice field area of Burkina Faso, West Africa. Medical and Veterinary Entomology, 26, 9–17. 21501199 10.1111/j.1365-2915.2011.00957.x PMC3140611

[37] Gómez GF, Márquez EJ, Gutiérrez LA, Conn JE, Correa MM. 2014. Geometric morphometric analysis of Colombian *Anopheles albimanus* (Diptera: Culicidae) reveals significant effect of environmental factors on wing traits and presence of a metapopulation. Acta Tropica, 135, 75–85. 24704285 10.1016/j.actatropica.2014.03.020 PMC4464773

[38] Govoetchan R, Sovi A, Aïkpon R, Salako A, Agbo FO, Asidi A, Akogbéto M. 2013. The impact of oviposition-site deprivation in gravid females of *Anopheles gambiae* (Diptera: Culicidae) on fecundity, trophic behaviour and life expectancy. International Journal of Tropical Insect Science, 33, 207–215.

[39] Gray E, Bradley T. 2005. Physiology of desiccation resistance in *Anopheles gambiae* and *Anopheles arabiensis**.* American Journal of Tropical Medicine and Hygiene, 73, 553–559. 16172480

[40] Gray EM, Rocca KAC, Costantini C, Besansky NJ. 2009. Inversion 2La is associated with enhanced desiccation resistance in *Anopheles gambiae**.* Malaria Journal, 8, 215. 19772577 10.1186/1475-2875-8-215 PMC2754996

[41] Hahn DA, Denlinger DL. 2007. Meeting the energetic demands of insect diapause: Nutrient storage and utilization. Journal of Insect Physiology, 53, 760–773. 17532002 10.1016/j.jinsphys.2007.03.018

[42] Hahn D, Denlinger D. 2011. Energetics of insect diapause. Annual Review of Entomology, 56, 103–121. 10.1146/annurev-ento-112408-085436 20690828

[43] Hidalgo K, Dujardin J-P, Mouline K, Dabiré RK, Renault D, Simard F. 2015. Seasonal variation in wing size and shape between geographic populations of the malaria vector, *Anopheles coluzzii* in Burkina Faso (West Africa). Acta Tropica, 143, 79–88. 25579425 10.1016/j.actatropica.2014.12.014

[44] Hidalgo K, Montazeau C, Siaussat D, Braman V, Trabalon M, Simard F, Renault D, Dabiré RK, Mouline K. 2018. Distinct physiological, biochemical and morphometric adjustments in the malaria vectors *Anopheles gambiae* and *Anopheles coluzzii* as means to survive dry season conditions in Burkina Faso. Journal of Experimental Biology, 221, jeb174433. 29378815 10.1242/jeb.174433

[45] Hidalgo K, Mouline K, Mamai W, Foucreau N, Dabiré KR, Bouchereau A, Simard F, Renault D. 2014. Novel insights into the metabolic and biochemical underpinnings assisting dry-season survival in female malaria mosquitoes of the *Anopheles gambiae* complex. Journal of Insect Physiology, 70, 102–116. 25083809 10.1016/j.jinsphys.2014.07.003

[46] Holstein M. 1954. Biology of *Anopheles gambiae.* Research in French West Africa. Geneva: World Health Organisation Monograph Series.

[47] Huestis DL, Artis ML, Armbruster PA, Lehmann T. 2017 Photoperiodic responses of Sahelian malaria mosquitoes *Anopheles coluzzii* and *An. arabiensis**,* Parasites & Vectors, 10, 621. 29282150 10.1186/s13071-017-2556-z PMC5745990

[48] Huestis DL, Lehmann T. 2014. Ecophysiology of *Anopheles gambiae* s.l.: persistence in the Sahel. Infection, Genetics and Evolution, 28, 648–661. 10.1016/j.meegid.2014.05.027 PMC4257857 24933461

[49] Huestis DL, Yaro AS, Traoré AI, Adamou A, Kassogué Y, Diallo M, Timbiné S, Dao A, Lehmann T. 2011. Variation in metabolic rate of *Anopheles gambiae* and *A. arabiensis* in a Sahelian village. Journal of Experimental Biology, 214, 2345–2353. 21697426 10.1242/jeb.054668 PMC3120220

[50] Huestis DL, Yaro AS, Traoré AI, Dieter KL, Nwagbara JI, Bowie AC, Adamou A, Kassogué Y, Diallo M, Timbiné S, Dao A, Lehmann T. 2012. Seasonal variation in metabolic rate, flight activity and body size of *Anopheles gambiae* in the Sahel. Journal of Experimental Biology, 215, 2013–2021. 22623189 10.1242/jeb.069468 PMC3359115

[51] Jaenson TG, Ameneshewa B. 1991. Prehibernation diet and reproductive condition of female *Anopheles messeae* in Sweden. Medical and Veterinary Entomology, 5, 243–252. 1768915 10.1111/j.1365-2915.1991.tb00547.x

[52] Jirakanjanakit N, Dujardin J. 2005. Discrimination of *Aedes aegypti* (Diptera: Culicidae) laboratory lines based on wing geometry. Southeast Asian Journal of Tropical Medicine and Public Health, 36, 858–861. 16295537

[53] Jirakanjanakit N, Leemingsawat S, Thongrungkiat S, Apiwathnasorn C, Singhaniyom S, Bellec C, Dujardin JP. 2007. Influence of larval density or food variation on the geometry of the wing of *Aedes (Stegomyia) aegypti**.* Tropical Medicine & International Health, 12, 1354–1360. 18045262 10.1111/j.1365-3156.2007.01919.x

[54] Krajacich BJ, Huestis DL, Dao A, Yaro AS, Diallo M, Krishna A, Xu J, Lehmann T. 2018. Investigation of the seasonal microbiome of *Anopheles coluzzii* mosquitoes in Mali. PLoS One, 13, 3 e0194899. 29596468 10.1371/journal.pone.0194899 PMC5875798

[55] Lee Y, Meneses CR, Fofana A, Lanzaro GC. 2009. Desiccation resistance among subpopulations of *Anopheles gambiae* s.s. from Selinkenyi, Mali. Journal of Medical Entomology, 46, 316–320. 19351082 10.1603/033.046.0216

[56] Lehmann T, Dao A, Yaro AS, Adamou A, Kassogue Y, Diallo M, Sékou T, Coscaron-Arias C. 2010. Aestivation of the African malaria mosquito, *Anopheles gambiae* in the Sahel. American Journal of Tropical Medicine and Hygiene, 83, 601–606. 20810827 10.4269/ajtmh.2010.09-0779 PMC2929058

[57] Lindsay SW, Parson L, Thomas CJ. 1998. Mapping the ranges and relative abundance of the two principal African malaria vectors, *Anopheles gambiae* sensu stricto and *An. arabiensis*, using climate data. Proceedings of the Royal Society of London. Series B: Biological Sciences, 265(1399), 847–854. 10.1098/rspb.1998.0369 PMC1689061 9633110

[58] Magnarelli L. 1979. Blood-feeding and gonotrophic dissociation in *Anopheles Punctipennis* (Diptera: Culicidae) prior to hibernation in Connecticut. Journal of Medical Entomology, 15, 278–281.

[59] Mala A, Irungu L, Mitaki E, Shililu J, Mbogo C, Njagi J, Githure J. 2014. Gonotrophic cycle duration, fecundity and parity of *Anopheles gambiae* complex mosquitoes during an extended period of dry weather in a semi-arid area in Baringo County, Kenya. International Journal of Mosquito Research, 1, 28–34.

[60] Mamai W, Mouline K, Blais C, Larvor V, Dabiré KR, Ouedraogo GA, Simard F, Renault D. 2014. Metabolomic and ecdysteroid variations in *Anopheles gambiae* s.l. mosquitoes exposed to the stressful conditions of the dry season in Burkina Faso, West Africa. Physiological and Biochemical Zoology, 87, 486–497. 24769712 10.1086/675697

[61] Mamai W, Simard F, Couret D, Ouedraogo GA, Renault D, Dabiré KR, Mouline K. 2016. Monitoring dry season persistence of *Anopheles gambiae* s.l. populations in a contained semi-field system in southwestern Burkina Faso, West Africa. Journal of Medical Entomology, 53, 130–138. 26576935 10.1093/jme/tjv174

[62] Masaki S. 1980. Summer diapause. Annual Review of Entomology, 25, 1–25.

[63] Minakawa N, Githure JI, Beier JC, Yan G. 2001. *Anopheline* mosquito survival strategies during the dry period in western Kenya. Journal of Medical Entomology, 38, 388–392. 11372963 10.1603/0022-2585-38.3.388

[64] Mouline K, Mamai W, Agnew P, Tchonfienet M, Brengues C, Dabire R, Robert V, Simard F. 2012. Physiology and development of the M and S molecular forms of *Anopheles gambiae* in Burkina Faso (West Africa). Medical and Veterinary Entomology, 26, 447–454. 22681446 10.1111/j.1365-2915.2012.01018.x

[65] Omer SM, Cloudsley-Thompson J. 1970. Survival of female *Anopheles gambiae* Giles through a 9-month dry season in Sudan. Bulletin of World Health Organisation, 42, 319–330. PMC2427450 5310144

[66] Omer SM, Cloudsley-Thompson J. 1968. Dry season biology of *Anopheles gambiae* Giles in the Sudan. Nature, 217, 879–880.

[67] Patz JA, Campbell-Lendrum D, Holloway T, Foley JA. 2005. Impact of regional climate change on human health. Nature, 438, 310–317. 16292302 10.1038/nature04188

[68] Reynolds J, Poelchau MF, Rahman Z, Armbruster PA, Denlinger DL. 2012. Transcript profiling reveals mechanisms for lipid conservation during diapause in the mosquito, *Aedes albopictus**.* Journal of Insect Physiology, 58, 966–973. 22579567 10.1016/j.jinsphys.2012.04.013 PMC3389261

[69] Rivero A, Agnew P, Bedhomme S, Sidobre C, Michalakis Y. 2007. Resource depletion in *Aedes aegypti* mosquitoes infected by the microsporidia *Vavraia culicis**.* Parasitology, 134, 1355–1362. 17634157 10.1017/S0031182007002703

[70] Robert V, Gazin P, Carnevale P. 1987. Malaria transmission in three sites surrounding the area of Bobo-Dioulasso (Burkina Faso): the savanna, a rice field, and the city. Bulletin of the Society of Vector Ecologists, 12, 541–543.

[71] Rocklöv J, Dubrow R. 2020. Climate change: an enduring challenge for vector-borne disease prevention and control. Nature Immunology, 21, 479–483. 32313242 10.1038/s41590-020-0648-y PMC7223823

[72] Roff D. 2002. Life history evolution. Sunderland, Massachusetts: Sinauer Associates, Inc.

[73] Santolamazza F, Mancini E, Simard F, Qi Y, Tu Z, Torre A. 2008. Insertion polymorphisms of SINE200 retrotransposons within speciation islands of *Anopheles gambiae* molecular forms. Malaria Journal, 7, 163. 18724871 10.1186/1475-2875-7-163 PMC2546427

[74] Simard F, Ayala D, Kamdem G, Pombi M, Etouna J, Ose K, Fotsing J-M, Fontenille D, Besansky NJ, Costantini C. 2009. Ecological niche partitioning between *Anopheles gambiae* molecular forms in Cameroon: the ecological side of speciation. BMC Ecology, 9, 17. 19460146 10.1186/1472-6785-9-17 PMC2698860

[75] Simard F, Lehmann T, Lemasson JJ, Diatta M, Fontenille D. 2000. Persistence of *Anopheles arabiensis* during the severe dry season conditions in Senegal: an indirect approach using microsatellite loci. Insect Molecular Biology, 9, 467–479. 11029665 10.1046/j.1365-2583.2000.00210.x

[76] Sinka ME. 2013. Global distribution of the dominant vector species of malaria, in Anopheles Mosquitoes – New Insights into Malaria Vectors. London, United Kingdom: InTechOpen. pp. 109–144.

[77] Stearns S. 1989. Trade-offs in life-history evolution. Functional Ecology, 3, 259–268.

[78] Tan Q, Feng L, Liu W, Zhu L, Lei C, Wang X. 2016. Differences in the pre-diapause and pre-oviposition accumulation of critical nutrients in adult females of the beetle *Colaphellus bowringi**.* Entomologia Experimentalis et Applicata, 160, 117–125.

[79] Tauber M, Tauber C, Masaki S. 1986. Seasonal adaptations of insects. New York: Oxford University Press.

[80] Tripet F, Touré YT, Taylor CE, Norris DE, Dolo G, Lanzaro GC. 2001. DNA analysis of transferred sperm reveals significant levels of gene flow between molecular forms of *Anopheles gambiae**.* Molecular Ecology, 10, 1725–1732. 11472539 10.1046/j.0962-1083.2001.01301.x

[81] Venkatesan P. 2025. WHO world malaria report 2024. Lancet Microbe, 6, 101073. 10.1016/j.lanmic.2025.101073 39923782

[82] Vinogradova EB. 2007. Diapause in aquatic insects, with emphasis on mosquitoes, in Diapause in Aquatic Invertebrates Theory and Human Use, Alekseev VR, De Stasio BT, Gilbert JJ, Editors. Springer, Netherlands: Dordrecht. pp. 83–113.

[83] Washino RK. 1977. The physiological ecology of gonotrophic dissociation and related phenomena in mosquitoes. Journal of Medical Entomology, 13, 381–388. 15116 10.1093/jmedent/13.4-5.381

[84] White GB. 1974. *Anopheles gambiae* complex and disease transmission in Africa. Transactions of the Royal Society of Tropical Medicine and Hygiene, 68, 278–298. 4420769 10.1016/0035-9203(74)90035-2

[85] Wiebe A, Longbottom J, Gleave K, Shearer FM, Sinka ME, Massey NC, Cameron E, Bhatt S, Gething PW, Hemingway J, Smith DL, Coleman M, Moyes CL. 2017. Geographical distributions of African malaria vector sibling species and evidence for insecticide resistance. Malaria Journal, 16, 85. 28219387 10.1186/s12936-017-1734-y PMC5319841

[86] Yaro AS, Traoré AI, Huestis DL, Adamou A, Timbiné S, Kassogué Y, Diallo M, Dao A, Traoré SF, Lehmann T. 2012. Dry season reproductive depression of *Anopheles gambiae* in the Sahel. Journal of Insect Physiology, 58, 1050–1059. 22609421 10.1016/j.jinsphys.2012.04.002 PMC4789105

